# Reducing radiation risks to staff for patients with permanently implanted radioactive sources requiring unrelated surgery

**DOI:** 10.1120/jacmp.v16i5.5372

**Published:** 2015-09-08

**Authors:** Parminder S. Basran, Patricia Baxter, Wayne A. Beckham

**Affiliations:** ^1^ Department of Physics and Astronomy University of Victoria Victoria BC; ^2^ Department of Medical Imaging Vancouver Island Health Authority Victoria BC Canada

**Keywords:** brachytherapy, prostate, radioactivity, seeds, risks

## Abstract

Permanent implant of sealed radioactive sources is an effective technique for treating cancer. Typically, the radioactive sources are implanted in and near the disease, depositing radiation absorbed dose locally over several months. There may be instances where these patients must undergo unrelated surgical procedures when the radioactive material remains active enough to pose risks. This work explores these risks, discusses strategies to mitigate those risks, and describes a case study for a permanent iodine‐125 (I‐125) prostate brachytherapy implant patient who developed colorectal cancer and required surgery six months after brachytherapy. The first consideration is identifying the radiological risk to the patient and staff before, during, and after the surgical procedure. The second is identifying the risk the surgical procedure may have on the efficacy of the brachytherapy implant. Finally, there are considerations for controlling the radioactive substances from a regulatory perspective. After these risks are defined, strategies to mitigate those risks are considered. We summarize this experience with some guidelines: If the surgical procedure is near (e.g., within 5–10 cm of) the implant; and, the surgical intervention may dislodge sources enough to compromise treatment or introduces radiation safety risks; and, the radioactivity has not sufficiently decayed to background levels; and, the surgery cannot be postponed, then a detailed analysis of risk is advised.

PACS numbers: 87.53Bn, 87.53Jw, 87.55.N, 87.56.bg

## I. INTRODUCTION

Permanent implant of sealed radioactive sources is an effective technique for treating cancer.^(1)^ Typically, the radioactive sources are implanted in and near the disease, depositing radiation absorbed dose locally over several months. Through strategic placement of the radioactive seeds in and around the tumor, local control of the disease can be achieved.

The handling of sealed radionuclides is subject to federal and sometimes local (provincial or state) regulations, as well as institutional policies and procedures. Generally, these regulations stipulate that radioactive seeds must be controlled before, during, and after placement inside the patient. Discharging a patient with radioactive seed implants from the hospital generally assumes that the seeds are secured inside the patient and have little or no chance of being released to the public. The risks to the public from a radioactive implant are assumed to be negligible when they are controlled within the patient, or are assumed not to have a total activity beyond a certain threshold.^2^, ^3^, ^4^ We define “active phase” for a radioactive seed (or a distribution of seeds) as the time between the brachytherapy procedure and the date at which the activity of the seeds is below its respective regulatory limit.

If radioactivity is released from the patient during its active phase, whether in a sealed state or not, there may be a nonnegligible risk to the general public, a nuclear worker (NW), or the patient. The risk to the general public has been explored in the context of deceased patients who have received permanent implant brachytherapy and have undergone cremation.^5^, ^6^, ^7^ A similar risk to health‐care workers, who may be members of the general public or NWs, may present itself if they attend to a patient who has radioactive implants in their active phase. In the course of their activities, health‐care workers may be exposed to radiation from a brachytherapy patient's implanted seeds if an unrelated surgery is required in an area close to the radioactive seed implants. For example, if a particular seed or a distribution of seeds in their active phase are removed or dislodged during an unrelated surgery, it is possible that the removed seed(s) could result in a breach of a regulatory‐equivalent or effective dose limit to members of public or a nuclear worker (NW).

In this situation, the safety and well‐being of the patient must also be considered. The purpose of the implants is to provide a therapeutic effect to a region of interest. A surgical intervention in an area close to the implants during the active phase could compromise the therapeutic intent of the implant.

This work explores the risk to health‐care workers and the patient if a surgical intervention is required in an area near permanent brachytherapy implants. We also briefly describe strategies to assess, monitor, and mitigate those risks, and provide a case study for a permanent I‐125 prostate brachytherapy implant patient who developed colorectal cancer and required surgery six months after implantation.

## II. MATERIALS AND METHODS

### A. Risk assessments

If the intended surgical procedure is near the brachytherapy implant such that: the dose‐rate to the surgical area is high; a surgical intervention may result in dislodging of seeds such that the treatment may be compromised; there is a risk through the course of the surgery of unwarranted radiation exposure to staff; the radioactivity has not sufficiently decayed to the specified background level (e.g., after two years for I‐125 or six months for palladium‐103); and the surgery date cannot be postponed to a date when the seeds are no longer considered to be in their active phase — then a more detailed analysis of risk may be in the best interest of the affected health‐care workers and patient.

Before any risk assessment can be done, a clear understanding of the surgical procedure is necessary. This would include understanding: the details of the standard procedure for surgical preparation, the surgery, and the postsurgical recovery; the possible use of radiopharmaceuticals or imaging equipment used during the procedure; methods used for incision and manipulation of tissues and organs; the extent of tissue to be removed; the method and extent of tissue removal and biopsied; standard procedures for postoperation recovery; and the number and vocations of staff involved in these procedures.

The risk of radiation exposure to involved health‐care workers may be estimated by calculating the equivalent and effective doses to staff from the implants due to the procedure. Whole body doses and equivalent doses (e.g., hands, skin, eyes), to staff may be estimated with information of the absorbed dose rate of the seed distribution, current activity, attenuating media between the sources and staff during the operation, expected duration of the surgery, and the positions of staff, hands, skin, and eyes (or other relevant organs or tissues) relative to the seeds within the operating room. Details on the location and distribution of the seeds, location of the surgical area, and current activity of the seeds can be obtained from patient imaging and knowledge of the implant.

The risk of compromising the brachytherapy treatment from the implants depends on the probability of dislodging or moving the seeds as a result of the procedure. The location of implants prior to the surgery can be assessed from an X‐ray computed tomography (CT) scan. These are typically acquired after a brachytherapy implant for evaluating the quality of the implant. From the CT scan, the distance between the surgical site and implants can be assessed. The risk of dislodging or moving the seeds can be assessed by obtaining a detailed understanding of the surgical procedure from the surgical team and the types of surgical tools used in the procedure, and an understanding of how parts of the anatomy are manipulated.

### B. Regulatory and local considerations

The regulatory requirements for situations where a patient has permanently implanted radioactive sources still in the active phase and requiring unrelated surgery are not entirely clear. There are a number of regulatory and site‐specific issues worth clarifying prior to surgery. One of the issues is whether the hospital performing the surgery will make provisions for the radiation safety of the patient and staff. It is quite possible that the hospital that administered the brachytherapy treatment is not the same as the hospital performing the unrelated surgery. Thus, the radiation safety officer (RSO) at the hospital where the surgery will be performed may not have sufficient information about the patient's implant to estimate the level of radiation risk to staff. Second, if there is a risk of exceeding annual effective or equivalent‐dose limits to staff attending to the patient, then regulatory bodies should be consulted. Third, if the surgery requires removal of seeds in their active phase, the transportation and storage of the seeds may be subject to both local and federal regulatory requirements. And finally, in the very unlikely scenario that the surgery could result in rupture of or contamination from the sealed sources, regulatory bodies may need to be consulted.^8^, ^9^ Given these considerations, documentation or a report of activities associated with the surgery may be required by local or federal regulatory bodies.

### C. Preoperation considerations

The operating room (OR) team generally consists of nurses and their assistants, the surgeon and assistant, anesthesiologists and their assistants, and other supporting individuals on the health‐care team, such as the cleaning and suite preparation staff. Depending on the degree of risk, the appropriateness of additional shielding for the OR team may need to be explored. It is important to appreciate that the OR team may have limited understanding of these risks and the strategies that might be used to minimize them. Education, communication, and careful planning are crucial.

If appropriate, a radiation safety education plan may need to be developed for the OR team. Some considerations in the education plan include: the use of time, distance and/or shielding of staff to reduce radiation exposure; the need to minimize the required staff in the surgical suite; the availability, location, and use of shielded containers (e.g., sterilized steel or lead‐lined trays) if sources or tissue containing sources are removed; the availability, location, and use additional radiation shielding in the suite, such as the use of lead aprons, portable lead‐glass shielding, lead‐lined gloves and glasses for all staff; and the appropriateness of dose monitoring, such as thermoluminescent dosimeter (TLD) rings, pocket dosimeters, and standard whole‐body dose badges by surgical staff.

In consultation with the OR team, a plan to monitor radiation exposure within the suite may be required. This may include surveying tissue samples taken from the patient before removal from the surgical suite; surveying linens, refuse, and surgical equipment before and after the surgical procedure; surveying catheters, catheter bags, and other such items if there is the possibility of seeds dislodging into the urethra/bladder; and monitoring the dose‐rate within the suite during the procedure, and real‐time dose‐monitoring of staff. A similar plan for monitoring radiation exposure in the postoperative recovery areas may be required. This may include surveying linens, refuse, and catheters prior to disposal, and educating staff on the need (if any) to implement the radiation protection principles of time‐distance‐shielding.

The radiation risks to all (OR and recovery room) staff from the surgical procedure may be estimated beforehand. Having knowledge of the expected equivalent and effective doses and expected dose‐rate before the procedure can be helpful in mitigating risks during the procedure.

Communicating with the surgeon well in advance may help gain an understanding of the surgical procedure and provide an opportunity to discuss radiation risks.

### D. Operation day considerations

A radiation safety procedure similar to those employed for a typical permanent implant brachytherapy procedure can be devised, with some notable exceptions, such as surveying for radioactive material within any removed tissue and vigilantly monitoring radiation levels during the OR procedure.

Depending on the estimated risk, staff may or may not be required to wear lead aprons, lead gloves, lead glasses, and TLD rings and/or pocket dosimeters. These needs should be balanced with the appropriateness of the shielding equipment and dosimeters given the duration and type of procedure. It is possible that some types of shielding may actually compromise the ability to perform the medical procedure effectively and so flexibility in radiation safety recommendations may be required. Background levels of radiation recorded in the OR suite before the surgery can provide a reference for subsequent measurements. The use of “Radiation Warning” signage at the OR entrance may help alert other staff not involved in the procedure.

Some surgical procedures require the use of radiopharmaceuticals to aid in the guidance of tumor excision, such as sentinel lymph node mapping^(10)^ or prolonged X‐ray usage. Care should be taken to ensure that radiation surveys are not confounded by other surgical procedures that may involve radiation. Surgical suites often have radiation barriers if portable diagnostic X‐rays or radiopharmaceuticals are used in the suite. Survey meters on very high sensitivity settings can detect emitted radiation from adjacent surgical suites. It may be necessary to ensure that such surgical procedures involving radiopharmaceutical use or prolonged X‐ray use are not performed close to the surgical suite, in order to ensure that the measurements of radiation are limited to those of the brachytherapy seeds.

Tracking the time from initial incision to final suturing/stitching will assist in estimating the equivalent and effective doses to the staff during the procedure. Continuously monitoring and recording the dose rate in the room during the surgery and warning staff of any anomalies will also have value. Finally, staff leaving the room during the procedure may need to be surveyed (e.g., surveying feet upon exiting the room) if there is a risk of contamination or seed removal.

If tissue is removed from the patient and there is a possibility that it contains radioactive material, a method for securing the radioactive material should be defined. This may be particularly important if the tissue sample is to be biopsied, where high precision microtomes could be damaged or, worse, rupture the radioactive seeds.

If tissue containing radioactive material is removed, the material should be stored in a suitable container with appropriate labeling (biohazard, radioactive material), subject to relevant regulations and in keeping with institutional radiation safety policies. This may imply handling of the material by an RSO or other appropriately trained individual, and the cataloguing and storage of the material until it can be safely disposed. All tissue removed from the patient can be easily scanned with a survey meter before disposal or removal from the surgical suite.

After the surgical procedure is complete, noting the total elapsed time of the procedure will help estimate the total equivalent and effective doses to the staff. In our facility, our standard operating procedure for brachytherapy includes surveying the OR team leaving the room to minimize the risk of contamination or a loose radioactive source in the room. Surveying all waste (lead gloves, catheters, disposable items), linens, the OR bed, surgical equipment, the floor, and anything else that may pose a risk of contamination is also performed. If the room is not contaminated, the background radiation level should be comparable with that based on readings taken before the OR procedure.

### E. Postoperation considerations

If personal dosimeters, such as TLD rings or badges are used, and there is reason to believe there are nonnegligible risks to the staff, effective and equivalent doses to all staff should be estimated as soon as possible after the surgery and appropriately followed up with regulatory bodies if necessary. If required, postoperative recovery areas may be surveyed for possible radiation sources. This would include scanning items such as linens, refuse, and catheters prior to disposal.

Radioactive material removed from the patient should be catalogued and safely stored in a shielded container, following regulatory requirements for storing radioactive material, such as identifying the radionuclide, number of seeds, date and activity, radiation signage, updating of sealed source inventory, and disposal date on the appropriately shielded container.

A postoperative CT scan provides an opportunity to record the number of seeds remaining in the patient, and an opportunity to evaluate the distribution of seeds to ensure the brachytherapy treatment has not been compromised. The brachytherapy‐implant physician may wish to contour the treatment volume and the surrounding normal tissues to assess whether or not the treatment remains therapeutically effective.

Finally, creating a report that summarizes the equivalent and effective doses to staff and the procedure details may be a valuable exercise to ensure that best practices were followed and that all risk factors have been minimized. Such a report may also act as a potential resource for similar events in the future, and may have educational value.

## III. RESULTS & DISCUSSION

### Case Study

### A. Risk assessments and pre‐OR considerations

We report a clinical situation where 85 I‐125 seeds were implanted in a cancer patient with a total activity of 35.4 U, intended to deliver 144 Gy to the prostate and adjacent extraprostatic area. The patient was then diagnosed with colon cancer only weeks after the implant. Surgery was delayed to 5.9 months after the implant date. The volume to be removed was the colon itself, approximately 2–4 cm from the posterior rectal volume containing radioactive seeds. Since portions of the rectum also required removal, there was a possibility of disturbing the seeds in the extraprostatic region at the base of the prostate, near the seminal vesicles. It was extremely unlikely the seeds themselves would be ruptured in the procedure given the types of instruments used in the surgery. The procedure entailed an abdominal incision, moving the bowel contents adjacent to the colon, removing the cancerous colon, obtaining a tissue sample near the rectum (for pathology), attaching the superior portions of the rectum with the inferior portions of the intestines, and suturing the incision.

Using the initial brachytherapy plan, implanted and current source activities, and a variety of CT scans, and assuming a duration of 2.5 hrs for the surgery, we estimated the effective doses to all staff and equivalent dose to hands to the surgeon and surgeon assistant in the procedure (Table 1). The surgeon elected to wear lead gloves but not to wear TLD rings due to the loss of dexterity. Based on the expected dose rate in the suite, the remaining staff was not required to wear additional protective clothing. The number of staff in the OR suite was minimized to two nurses, the surgeon, surgeon's assistant, anesthesiologists, and a medical physicist. The surgical suite with the largest footprint was booked in order to take advantage of time‐distance dose reductions to staff. The use of distance was applied, along with vigilant monitoring of dose rate and equivalent dose with survey meters and electronic personal dosimeters. All staff wore their standard‐issue TLD body badges.

**Table 1:** Estimates of effective and equivalent dose and limits for radiation workers (RWs) and the general public. The maximum dose rate measured was above the patient's abdomen (0.4 μSv/hr), and the maximum measured total dose to any staff was 1 μSv/hr

*Dose at Distance from Brachytherapy Implant Area*	*Expected Dose Rate* (μSv/hr)	*Expected Dose (mSv)*
Staff @ 1 meter with no patient shielding	$9.5^{a}$	$0.04^{a}$
Surgeons @ 30 cm		0.12
hands without Pb gloves @ 10 cm	–	1.12
@ 1 cm		112.3
Surgeons @ 30 cm		0.04
hands with Pb gloves @ 10 cm	–	0.39
@ 1 cm		39.3

a The annual equivalent dose limit is 50 mSv in Canada and the United States, or approximately 25 μSv/hr over a 40‐hour work week. The expected dose calculation assumes a 2.5‐hour surgery duration.

### B. Regulatory considerations

There was effectively no risk of seeds being ruptured. If potentially contaminated biohazardous tissue containing seeds were removed from the patient, large and small lead‐lined containers were kept close by the operating table. It was also confirmed that the institution's license enabled possession, use, and storage of the radionuclide of concern.

### C. Operating and postoperating day considerations

**Figure 1:**
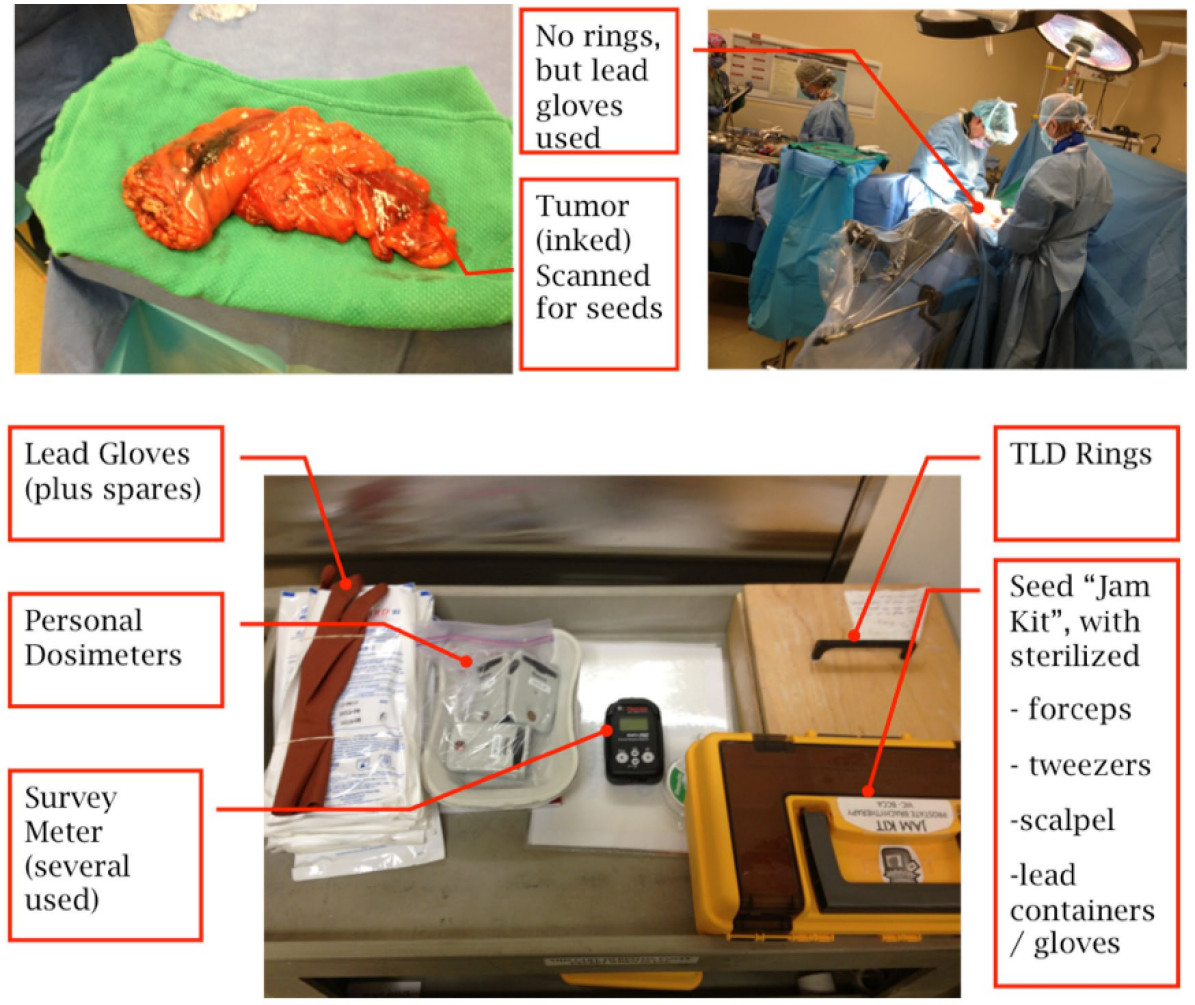
Photographs of the removed colorectal volume (upper left), the surgical procedure (upper right), and the surgical kit (bottom). Not shown are lead glasses, stopwatch, lead containers (for sources only), lead envelope (shielding material), and large metallic container for holding/transporting biohazard/radioactive material.

Standard operating procedures for a brachytherapy implant were used during the surgery, but with a few exceptions. Personal dosimeters, lead gloves, and lead aprons were used for the surgeon and assistant. Another dosimeter was placed adjacent to the patient for additional monitoring. Nursing and anesthesiology staff elected to wear lead aprons, but were not required to do so from a radiological risk perspective (see Fig. 1).

The entire procedure lasted a little over 2 hrs. The dose rate at 1 meter from the patient was at or slightly above background levels (0.03−0.05 μSv/hr). The maximum dose rate measured was approximately 0.4 μSv/hr, at approximately 30 cm from the patient just above the abdominal cavity. This was measured for approximately 30 min mid‐way through the surgery when the bowel contents were moved adjacent to the colon when there was the least amount of tissue between the seeds and staff. Tissue samples (cancerous region plus a tissue sample 2 cm from the tumor) were surveyed, and radiation levels were no higher than background (0.03 μSv/hr). Table 1 summarizes estimates and measurements of effective and equivalent doses in the procedure. Immediately after surgery, the room, garbage, linens, and equipment were scanned and all measurements were no higher than background. The maximum whole‐body dose measured from personal dosimeters was 1 μSv/hr for the surgeon's assistant, who spent the longest period of time next to the patient. The measured whole‐body dose for all other staff was less than 1 μSv/hr. Immediately after the surgery, the dose rate above the surgical site on the patient's abdomen was less than 0.4 μSv/hr. This measurement point was approximately 10 cm from the location of the seeds within the patient.

**Figure 2:**
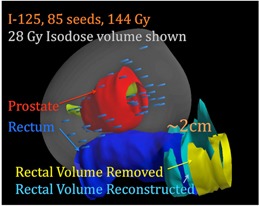
Three‐dimensional rendering of the prostate, prostate seeds, presurgical rectum, and the postsurgery reconstructed rectum.

A CT scan of the pelvis was acquired several hours after the surgery and the CT dataset was imported into a brachytherapy treatment planning workstation. The postsurgery CT scan was registered with the original brachytherapy plan and the implanting physician contoured the prostate, rectum, bladder, and urethra on the postsurgery CT. The brachytherapy software detected 85 seeds in the postsurgery CT set which was equal to the number of seeds in the CT scan before the surgery; this suggests that no tissue containing seeds was removed from the patient. The seed positions, prostate, bladder, and urethra tissue contours, and the radiation absorbed dose from the seeds to the prostate and normal tissues, could be directly compared with those from the postimplant CT scan. While there were some shifts in individual seed positions, the dose to the prostate, bladder, and urethra was not deemed clinically different from the original plan, and the original therapeutic intent of the implants was not affected as a result of the surgery. Because the surgery required significant removal of the patient's colorectal volume, there were observable differences in the rectum position (see Fig. 2).

The patient experienced minimal blood loss, and urine and fecal discharges during their stay in the in‐patient ward. Linens and garbage were surveyed after the first day with no seeds detected. A report was provided to all staff on the effective and equivalent doses, and dose rates from the procedure, as obtained from pocket dosimeters and survey meters. The report was subsequently used in developing a generalized strategy for the hospital.

## IV. CONCLUSIONS

If a surgical procedure is required in a region near brachytherapy implants that are in their active phase, there may be undue negative consequences to the surgical staff and the patient. Only through a clear understanding of the surgical procedure and consultations with the surgical team can the risks be fully appreciated. By establishing an educational plan and by carefully planning preoperation, operation, and postoperation recovery, the risks to health‐care workers — and the patient — can be assessed, mitigated, and eliminated.

## ACKNOWLEDGMENTS

The authors would like to thank Dr. Howard Pai, MD, Dr. Bao Tang, MD, Petra Hrasky R.N., Siobhan Doyle R.N., and Susan Rombout R.N. for their helpful discussions.
